# Chemoradiotherapy for Inoperable Carotid Body Leiomyosarcoma: A Case Report and Review of Literature

**DOI:** 10.3389/fonc.2020.599403

**Published:** 2021-02-11

**Authors:** Cheng-Sheng Liu, Jia-Ruey Tsai, Yi-Tzu Kao, Long-Sheng Lu, Yin-Ju Chen, Thierry Burnouf, Peng-Yuan Wang, Jeng-Fong Chiou, Lai-Lei Ting

**Affiliations:** ^1^ Department of Radiation Oncology, Taipei Medical University Hospital, Taipei, Taiwan; ^2^ Division of Hematology and Oncology, Department of Internal Medicine, Taipei Medical University Hospital, Taipei, Taiwan; ^3^ Graduate Institute of Biomedical Materials & Tissue Engineering, College of Biomedical Engineering, Taipei Medical University, Taipei, Taiwan; ^4^ International Ph.D. Program in Biomedical Engineering, College of Biomedical Engineering, Taipei Medical University, Taipei, Taiwan; ^5^ Centre for Human Tissue & Organs Degeneration, Shenzhen Institutes of Advanced Technology, Chinese Academy of Sciences, Shenzhen, China; ^6^ Department of Chemistry and Biotechnology, Swinburne University of Technology, Hawthorn, VIC, Australia; ^7^ Taipei Cancer Center, Taipei Medical University, Taipei, Taiwan; ^8^ Department of Radiology, School of Medicine, College of Medicine, Taipei Medical University, Taipei, Taiwan

**Keywords:** case report, leiomyosarcoma, carotid body tumor, concurrent chemoradiotherapy, liquid biopsy

## Abstract

Vascular leiomyosarcoma is an extremely rare tumor and is associated with poor prognosis among leiomyosarcoma. Surgical resection remains the main treatment option. But outcome of definitive treatment with chemoradiotherapy in inoperable patients is not clear. Here, we report treatment and outcome of definitive chemoradiotherapy in a case of vascular leiomyosarcoma. A 64-year-old man with the initial presentation of pulsatile right neck mass was diagnosed with right carotid body leiomyosarcoma. He refused surgical intervention due to risk of carotid body injury and ischemic stroke. Successful tumor control was achieved with carboplatin-based concurrent chemoradiotherapy. Investigational liquid biopsy for circulating sarcoma cells was also performed to analyze drug sensitivity profile of this rare tumor. One year after treatment, the disease remained well controlled and there was no evidence of baroreflex failure or treatment-related late toxicities. To our best knowledge, this is the first case report of right carotid body leiomyosarcoma controlled with definitive concurrent chemoradiotherapy. The approach of personalized multi-modality treatment will be a focus of our future investigation.

## Introduction

Sarcomas are a rare type of malignancy that account for less than 1% of all adult cancers. More than 80% of sarcomas originate from soft tissue, with the remaining, less than 20%, of them arising from bone. Soft tissue sarcomas are a heterogenous group of cancers that originate from the embryonic mesoderm and can originate in any part of the body. It usually originates in extremities but can sometimes occur in the trunk, retroperitoneum, or the head and neck region. The most common subtype of soft tissue sarcomas is liposarcoma, undifferentiated/unclassified soft tissue sarcoma, followed by leiomyosarcoma (LMS). LMS comprise 12% of all soft tissue sarcomas, which usually arise from the smooth muscle layers of uterus, gastrointestinal tract, intra-abdomen origin, retroperitoneum, and rarely the blood vessels (1). Primary vascular LMS is an extremely rare tumor that presents as palpable masses leading to arterial occlusion, venous stasis, or nerve compression. These tumors grow slowly and are often misdiagnosed as atherosclerotic chronic occlusive disease or aneurysm if presented with palpable pulsatile mass. Vascular LMSs more commonly affect veins than arteries and the majority originate from the inferior vena cava (IVC) (2). As compared with LMS of other origin, vascular LMS have significantly shorter metastasis-free survival and overall survival, 0.25 years and 2.1 years respectively (3).

Despite a lack of high-level evidence, surgical resection is the mainstay of treatment for these patients. The role of chemotherapy and/or radiotherapy (RT) for vascular LMS remains inconclusive. Clinical descriptions are limited to single case report or case series for vascular LMS of the arterial origin. Due to its rare incidence and poor prognosis, personalized treatment is challenging to multidisciplinary tumor board (MDT). Here, we report our experience of treating one case with primary arterial LMS arising from the right carotid body. Disease control was achieved with carboplatin-based concurrent chemoradiotherapy (CCRT). Relevant literature review on peripheral arterial LMS is also provided.

## Case Presentation

**Figure 1:**
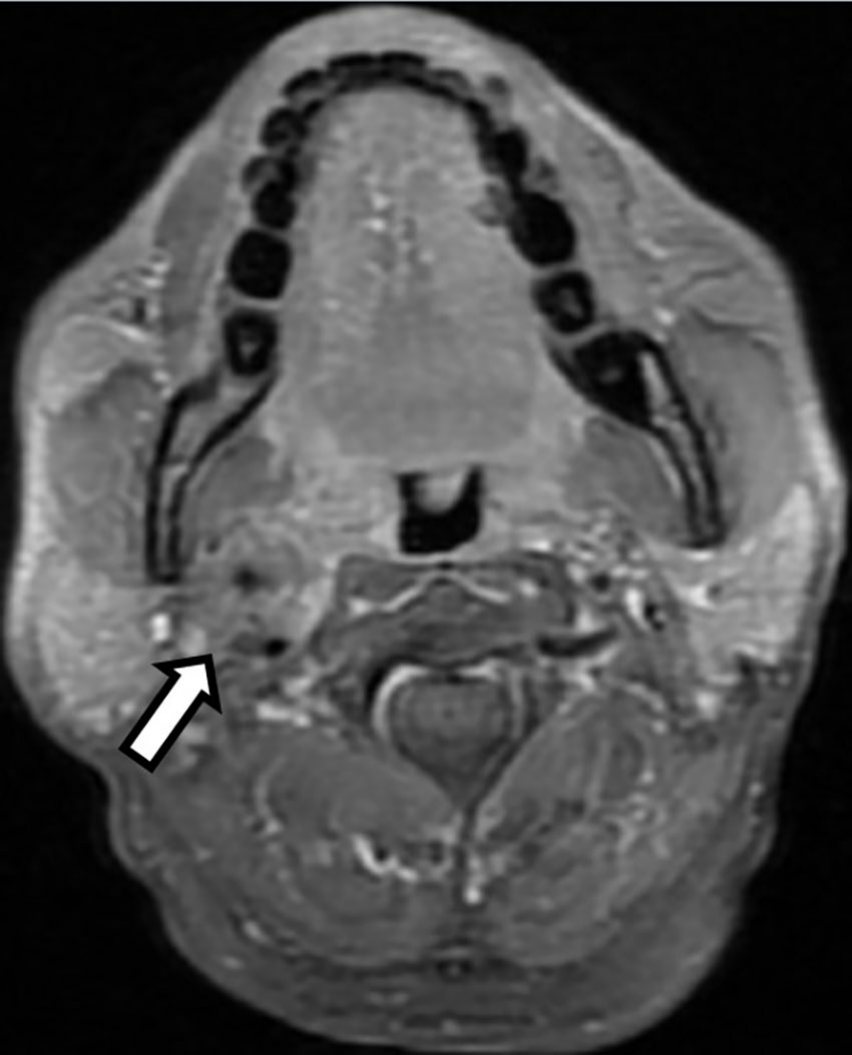
T1-weighted head and neck magnetic resonance image (MRI) with fat saturation revealed a 2.6 centimeter enhancing soft tissue lesion occupying the right carotid space and the right parapharyngeal space at the level of carotid bifurcation with right internal and external carotid artery encasement (Open arrow).

A 64-year-old male presented to the oncology department of our hospital with pulsatile right neck mass for one year. It was associated with dizziness and near-syncope. He was quite healthy without past medical history. On physical examination, a pulsatile mass about 2 cm was palpable over right submandibular region. Carotid bruit was not audible while auscultation and the mass were invisible upon oral cavity inspection. The absence of symptoms of hoarseness or dysphagia indicated no pressure on the larynx or pharynx. Vocal cord paralysis was also excluded after laryngoscopy examination. Magnetic resonance image (MRI) of the head and neck revealed a 2.6-centimeter soft tissue mass occupying the right carotid space and the right parapharyngeal space at the level of carotid bifurcation (**Figure 1**). The mass encased and constricted both the external and internal carotid arteries. The differential diagnosis included early nasopharyngeal mucosal lesion with submucosal spreading, metastatic lymphadenopathy, and neurogenic tumor. Contralateral carotid body was free of disease and there were no regional lymphadenopathy or distant metastases on computed tomography as well as whole-body bone scan.

He received biopsy of the carotid body tumor. Pathology reported a high grade leiomyosarcoma with non-limited positive margins. Immunohistochemically, tumor cells are positive for desmin, actin, and h-caldesmon and negative for ER, S-100, CD34, and beta-catenin. Therefore, a diagnosis of right carotid body leiomyosarcoma, high grade, stage IIB was made. After thorough discussion in MDT, platinum-based CCRT was recommended.

**Figure 2:**
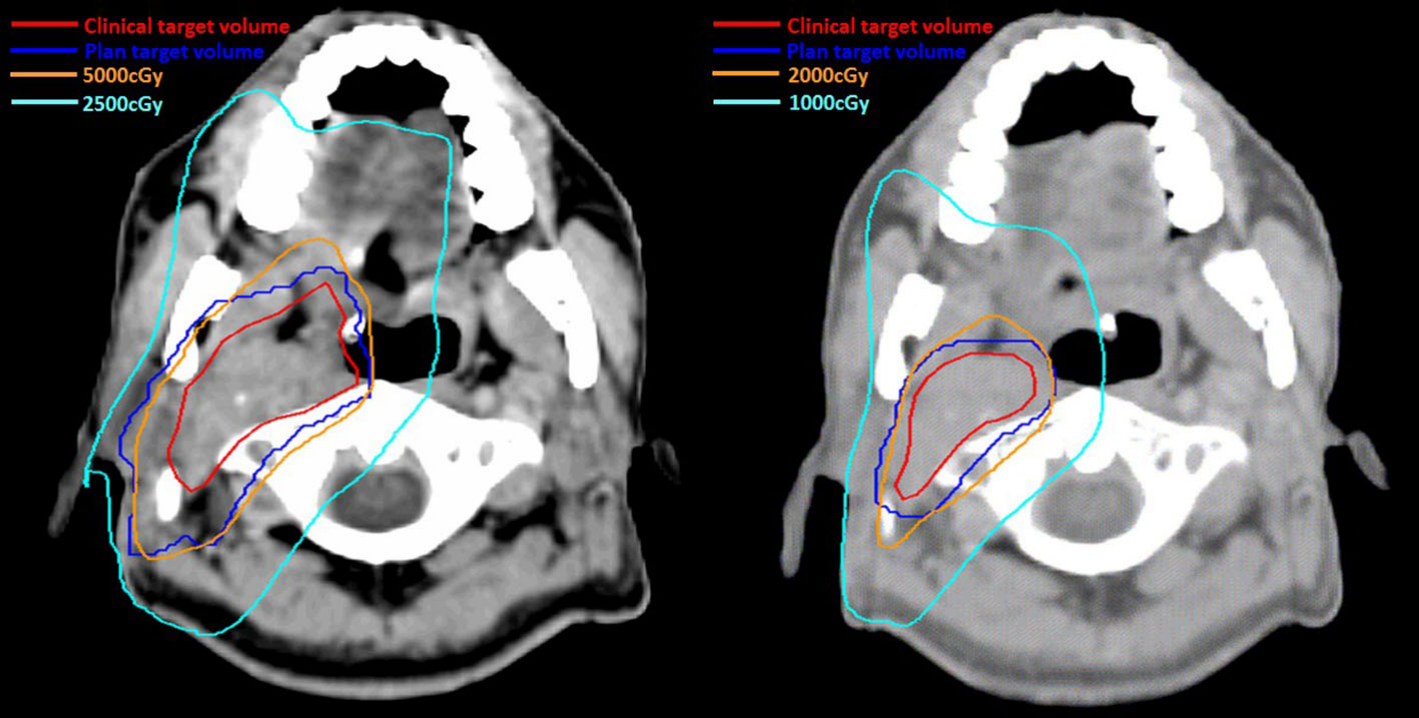
Irradiation 50Gray (Gy) in 25 fractions (Fx) was delivered to carotid body tumor and right neck regional lymphatics (level Ib to level III) (Left) followed by boost radiotherapy 20Gy in 10Fx to carotid body tumor (Right). The red and blue lines represented the clinical target volume and plan target volume respectively. The orange line and light blue lines represented the isodose line of the 100% and 50% prescribe dose in two phases of radiotherapy, respectively.

**Figure 3:**
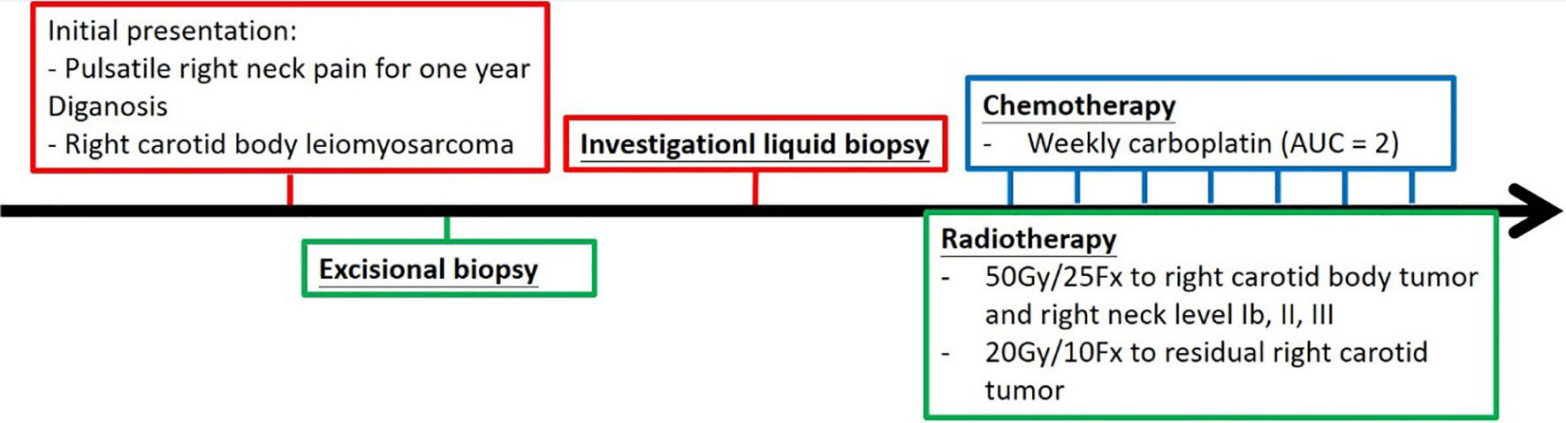
Treatment course of this case was represented in a timeline. Weekly dose of carboplatin (area under curve 2) was administrated every week for 6 cycles, and conventional fractionated radiotherapy to carotid body and regional lymphatics was delivered concurrently with chemotherapy.

**Figure 4:**
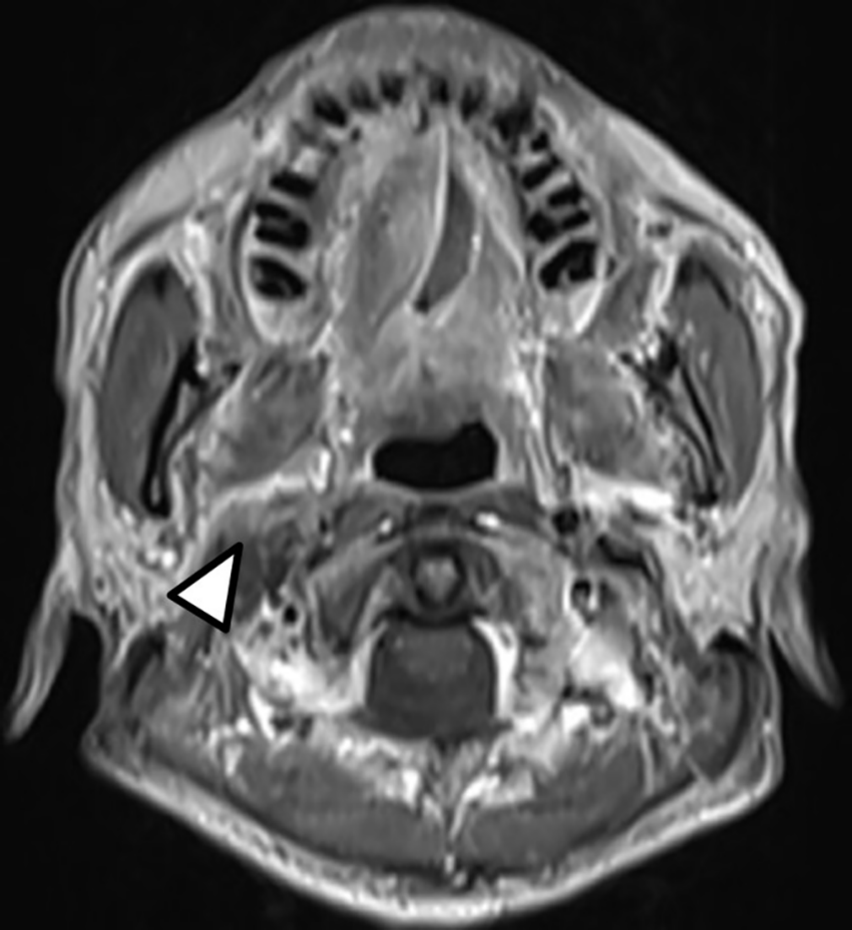
Post-treatment T1-weighted head and neck MRI showed decreased size of the right carotid body tumor with slightly anterior displacement of the right parapharyngeal fat (Arrow head).

Definitive carboplatin-based CCRT was then initiated one month after biopsy. Conventional fractionated RT to right carotid body tumor and right neck level Ib, II, III (50 Gray [Gy] in 25 fractions [Fx]) followed by right carotid tumor boost (20 Gy in 10 Fx) was delivered *via* 6MV photons (**Figure 2**). Weekly carboplatin (area under curve = 2) was administrated concurrently with RT for six cycles. An episode of hospital acquired pneumonia happened during the first month of treatment and subsided after antibiotic treatment. Acute side effects of grade 2 skin reaction, grade 2 oral mucositis, and grade 1 cervical esophagitis were observed during treatment course and were medically controlled without treatment interruption (**Figure 3**). Post-treatment MRI study revealed decreased main tumor size three months after CCRT (**Figure 4**). He did not receive adjuvant chemotherapy, and the disease remained controlled one year after the treatment without treatment related late toxicities. In addition, his blood pressure is within normal range and there was no evidence of postural hypotension or episodes of syncope or near syncope. Owing to the absence of symptoms of baroreflex failure, neurophysiology examinations are not indicated for this patient.

**Figure 5:**
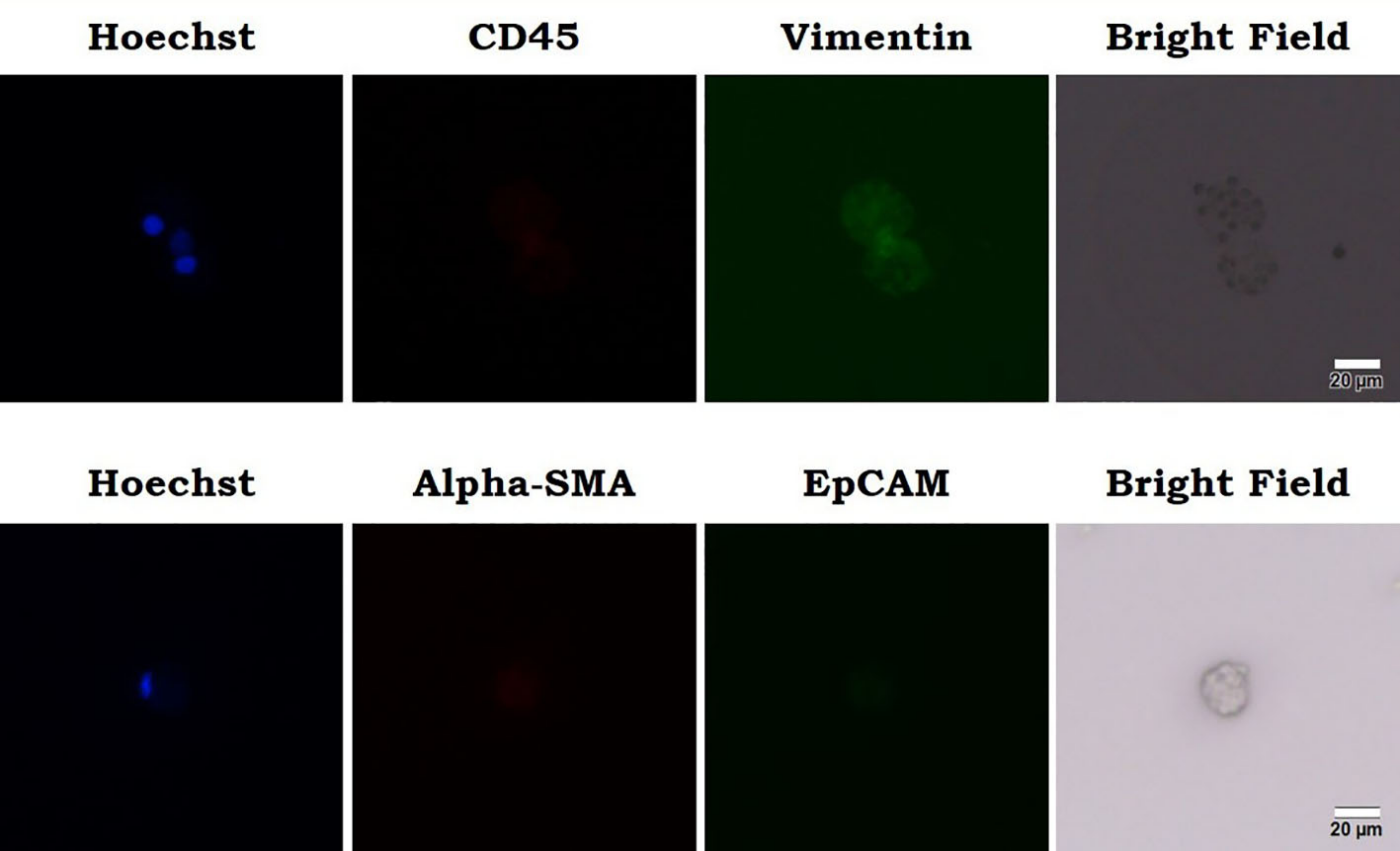
Outgrowth cellular organoids were observed and cells were stained positive for vimentin and negative for CD45 or EpCAM. Cellular sensitivity to cytotoxics was measured with relative ATP abundance comparing to cells treated with mock solvent.

**Figure 6:**
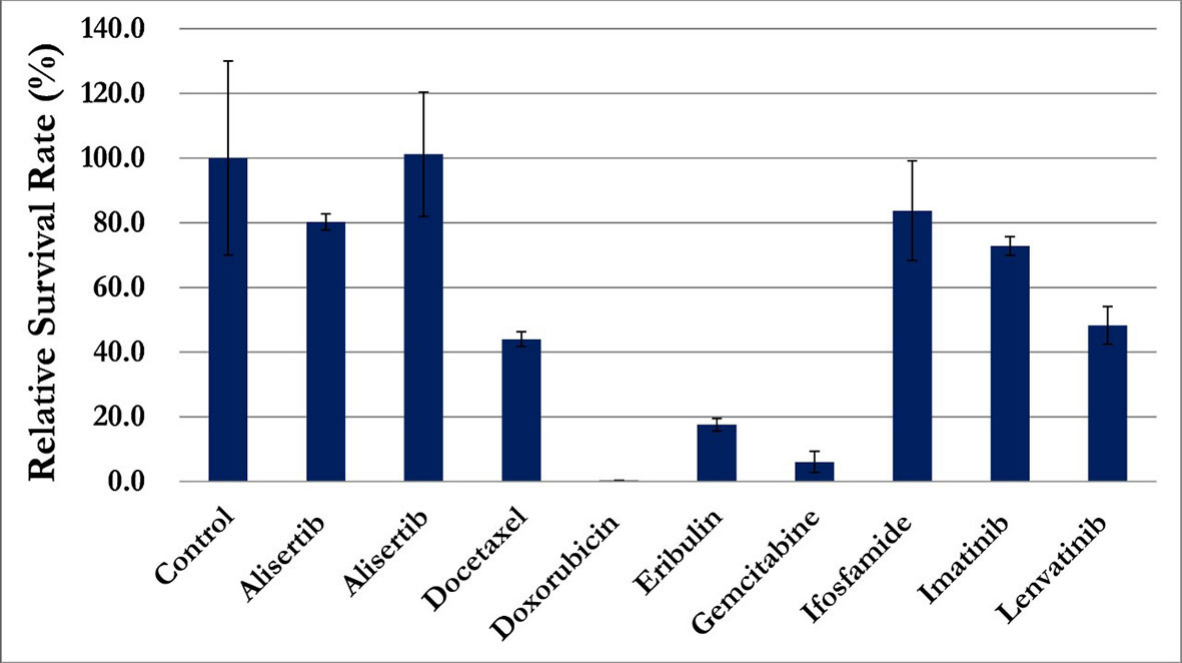
Proliferation suppression of circulating sarcoma cells, more than 80% of growth inhibition, was observed for doxorubicin, eribulin, gemcitabine, olaratumab/doxorubicin combination, and pazopanib.

In addition to routine clinical workup, an investigational liquid biopsy to analyze circulating sarcoma cells (CSC) was performed if salvage treatment indicated after CCRT. Peripheral venous blood sample was processed for mononuclear cell fraction and was cultured in a binary colloid crystal-coated microfluidic chip to allow clonal expansion. The outgrowth cellular organoids were observed after 4 weeks and cells were stained positive for vimentin and negative for CD45 or EpCAM (**Figure 5**). Furthermore, cellular sensitivity to cytotoxics was measured with relative ATP abundance comparing to cells treated with mock solvent. More than 80% proliferation suppression was observed for doxorubicin, eribulin, gemcitabine, olaratumab/doxorubicin combination, and pazopanib at the equivalent concentration of Cmax corresponding to a standard treatment dose (**Figure 6**).

## Discussion

Management of soft tissue sarcoma is challenging to clinical physicians due to its rare incidence, heterogeneity of natural history, and unpredictable response to treatment owing to diverse histology. In addition, special considerations were needed for site-specific tumors. For adult localized disease, surgical resection with appropriate margins is the standard treatment of all adult sarcoma patients. Regional lymph node dissection is not routinely required because the risk of metastatic lymphadenopathies is quite low. However, surgical measure is not always practicable due to the limitations of critical anatomical structures such as major nerve and blood vessels. In addition, positive margins through microscopic examination or macroscopic residual disease imply high risk of local recurrence. Adjuvant treatment, including adjuvant RT, is mandated for microscopic or macroscopic diseases and is associated with improving local control rate from 56% to 74% (4).

Among head and neck cancers, soft tissue sarcomas are rare and only take 1% among malignancy in the head and neck region. LMSs of the head and neck region can be derived from either cutaneous or subcutaneous origin. Surgical rection is the main treatment options for LMSs of head and neck origin and RT is reserved for inoperable cases or patients’ preferences. LeVay et al. reported a single institute treatment experience for soft tissue sarcoma, including LMSs of the head and neck origin (5). As compared with sarcomas of other origin, sarcomas of the head and neck origin are associated with higher 5-year local relapse rate of 41% and worsened 5-year overall survival (OS) of 63%. In population-based analyses, Eppsteiner et al. reported the 5-year OS for 578 patients with LMSs over head and neck region was 56.4%, which was similar to cases previously reported (6). In addition, several clinical pathological factors, including low tumor grade of primary lesions, LMSs of cutaneous origin, and negative surgical margins, are associated with better disease specific survivals (7, 8).

Vascular LMSs are rare, taking up less than 5% of all LMSs, and involved veins more often than arteries (9). Half of the cases were originating from IVC. Roland et al. reported 63 patients with primary localized vascular LMSs between 1993 and 2012. All of the tumors arise from venous systems with two-thirds of the tumors arising from IVC. The results showed that the 5-year disease specific survival of 65% following surgical resection. Molecular analysis was also performed retrospectively in the study and found that expressions of beta-catenin and insulin like growth factor 1 receptor were associated with inferior disease-specific survival (10). Arterial LMSs are less common with more aggressive behavior and inferior outcomes as compared with venous LMSs. Symptoms of arterial leiomyosarcoma are associated with intraluminal occlusion and can vary widely according to the location of the tumor. Therefore, misdiagnosed and delayed diagnosis occasionally happened owing to similar presenting symptoms with peripheral arterial occlusive disease or aneurysm. Arterial LMSs usually affect the heart and great vessels including pulmonary arteries, thoracic or abdominal aorta, followed by lower extremities vessels. **Table 1** lists the details including location, histology, and treatment modalities of previous case reports of LMSs originating from peripheral arteries other than heart, great vessels, and lower extremities (11–15). Treatment of arterial LMS mainly rely on surgical resection with negative margins and vascular reconstruction often required owing to absence of collateral vessels. However, the feasibility of surgical intervention mainly depends on the location of tumor.

**Table 1:** Summary of published case reports and case series on rare origin arterial leiomyosarcoma (LMS).

Case No.	1	2	3	4	5
Year	1977	1995	2000	2010	2019
Sex	Female	Male	Female	Female	Male
Age	37	51	76	67	63
Location	Internal mammary artery	Subclavian artery	Renal artery	Brachial artery	Superior mesenteric artery
Initial presentation	Anterior mediastinal mass	Right shoulder and back pain	Left renal hilum mass	Mid-upper left arm lump	Epigastric pain
Treatment modalities	Tumor excision	Tumor excision + adjuvant radiotherapy	Left radical nephroadrenalectomy	Tumor excision	En-bloc resection + adjuvant chemotherapy
Tumor size	Not available	8.0cm	10cm	3.0cm	4.2cm
Grade	Not available	High	High	Intermediate	Not available
Follow-up period	Not available	12 months	9 months	15 months	6 months
Recurrence	No	Yes (Local, 10 months)	No	No	No
Survival	Not available	No (12 months)	Yes	Yes	Yes
References	(11)	(12)	(13)	(14)	(15)

Tumors arising from the carotid body are extremely rare with the incidence of less than 1 in 30,000 and are exclusively benign or malignant paraganglioma in histology. Carotid body is a chemoreceptor located in the adventitia over external carotid body near the bifurcation of common carotid artery. It monitors the oxygen tension of systemic arterial blood and modulates cardiovascular and respiratory systems through sympathetic tone. Given the importance of chemoreceptor cells in the carotid body, resection remains risky for carotid body tumor and harbors the possibility of injury to cranial nerves, carotid arteries, and excessive blood loss. Therefore, radical surgical intervention with vascular reconstruction was not recommended after otolaryngologist consultation for this patient with carotid body LMS. In this circumstance, definitive radiotherapy after biopsy with positive margins provide effects on local control. Although low risk of regional nodal involvement in LMS, Yadav et al. reported late lymph node metastases in 10% of patient with head and neck LMS (16). Given the risk of regional recurrence and re-irradiation to the same field may not be feasible, the regional lymphatic was also included in the radiation field. Beside surgical intervention, irradiation to head and neck region also carry the risk of injury to carotid body. Sharabi et al. reported three cases of head and neck cancers that encountered baroreflex failure as a possible late complication several years after CCRT (17). In our case, there were no orthostatic hypotension, nor syncope episode observed one year after CCRT. There was lack of evidence in adding chemotherapy concurrently or sequentially with radiotherapy for vascular leiomyosarcoma. In two large cancer center experiences, systemic chemotherapy is recommended for head and neck soft tissue sarcomas with features of high risk of recurrence (high grade, deep location, and size > 5cm) (8, 18).

Soft tissue sarcomas have risk of recurrence despite initial curative treatment. Patients with vascular LMS had a significantly worse median metastasis-free survival and overall survival (0.25 years and 2.1 years respectively) as compared with LMS of other origin (9.6 years and 7 years) (3). Anthracycline-based chemotherapy regimen was commonly administrated as salvage treatment. Despite significant chemotherapy related toxicities, the response rate was approximately 30% and the median overall survival was approximately one year (19, 20). Precision medicine can provide treatment options for individual patients with various histology and its unique tumor behavior. Therefore, investigational liquid biopsy was performed before definitive treatment in case of salvage systemic anti-cancer treatment indicated. CSCs were expanded and analyzed for putative drug sensitivity profile prior to treatment. Interestingly, these cells responded to many of the classical agents for sarcoma treatment, including doxorubicin, eribulin, and pazopanib. Moreover olaratumab, a monoclonal antibody to PDGFR-alpha, did not show meaningful activity. This is consistent with the ANNOUNCE trial that adding olaratumab fails to demonstrate overall survival benefit over standard treatment for patients with soft tissue sarcoma (21). In the era of precision oncology, such an approach has potential to provide personalized data and scientific evidence to support treatment decisions in the absence of high-quality clinical evidence for patients beyond standard treatment.

To our best knowledge, this is the first case report of vascular LMS arising from the right carotid body. Owing to its complexity, the patient received multimodality treatment with carboplatin-based CCRT instead of radical surgery after comprehensive discussion in head and neck MDT. The disease remained controlled after one year of surveillance. The patient lives with preserved quality of life and is satisfied with excellent tumor control during this period. Moreover, investigational liquid biopsy with CSC and molecular and cellular analysis demonstrated potential for personalized drug sensitivity profiling. Although CCRT is associated with promising tumor control, it is not an evidence-based approach owing to the rare disease entity. Furthermore, there are no detailed neurophysiological examinations to clarify neurological function despite the absence of symptoms of baroreflex failure. In conclusion, definitive CCRT is well tolerated and achieved promising tumor control for this patient with right carotid body LMS. Further investigation will be necessary for treating inoperable peripheral arterial LMSs with definitive CCRT.

## Data Availability Statement

The raw data supporting the conclusions of this article will be made available by the authors, without undue reservation.

## Ethics Statement

The studies involving human participants were reviewed and approved by Office of Human Research, Taipei Medical University. Written informed consent to participate in this study was provided by the participants’ legal guardian/next of kin.

## Author Contributions

Conceived and designed the experiments: L-SL, J-FC, and L-LT. Analyzed the data: C-SL, J-RT, Y-TK, L-SL, Y-JC, TB, and P-YW. Contributed reagents/materials/analysis tools: L-SL, Y-JC, TB, and P-YW. Critical discussion and reading: J-FC and L-LT. Wrote the paper: C-SL, J-RT, L-SL, and L-LT. All authors contributed to the article and approved the submitted version.

## Funding

This project is supported by the National Health Research Institutes, Ministry of Science and Technology, and Taipei Medical University (Grants NHRI-EX108-10713E1, USTP-NTUT-TMU-105-06, USTP-NTUT-TMU-106-01, USTP-NTUT-TMU-107-01, 108-2314-B-038 -133-MY1, 108-2314-B-038-038-MY1, 105TMU-CIT-02-2, 106TMU-CIT-02-2, TMU104-AE1-B27).

## Conflict of Interest

The authors declare that the research was conducted in the absence of any commercial or financial relationships that could be construed as a potential conflict of interest.
